# Microbial Diversity Drives Decomposition More than Advantage of Home Environment—Evidence from a Manipulation Experiment with Leaf Litter

**DOI:** 10.3390/microorganisms13020351

**Published:** 2025-02-06

**Authors:** Masoud M. Ardestani, Jaroslav Kukla, Tomáš Cajthaml, Petr Baldrian, Jan Frouz

**Affiliations:** 1 Institute for Environmental Studies, Charles University, Benátská 2, 12801 Prague, Czech Republic; mortazavi_m2000@yahoo.com (M.M.A.); jaroslav.kukla@natur.cuni.cz (J.K.); cajthaml@biomed.cas.cz (T.C.); 2 Institute of Soil Biology and Biogeochemistry, Biology Centre of the Czech Academy of Sciences, Na Sádkách 7, 37005 České Budějovice, Czech Republic; 3 Institute of Microbiology of the Czech Academy of Sciences, Vídeňská 1083, 14200 Prague, Czech Republic; baldrian@biomed.cas.cz

**Keywords:** bacteria, decomposition of soil organic matter, fungi, microbial biomass, microbial diversity, succession

## Abstract

Microbial diversity plays a crucial role in litter decomposition. However, the relationships between microbial diversity and substrate successional stage are the drivers of this decomposition. In this study, we experimentally manipulated microbial diversity and succession in post-mining soil. We used leaf litter samples from two forests of a post-mining site near Sokolov, Czech Republic: one alder plantation and one mixed forest with birch aspen and willow. Litter from each site was decomposed in the field for 3 and 12 months. The litter was X-ray sterilized and part of the litter was kept unsterilized to produce inoculum. Leaf litter samples of two different ages (3 and 12 months) from each site were each inoculated with litter of two different ages (3 and 12 months), using less and more diluted inoculum, producing two levels of microbial diversity. In each of these eight treatments, the bacterial community was then characterized by amplicon sequencing of the 16S rRNA gene and microbial respiration was used to assess the rate of decomposition. A significantly higher respiration (*p* < 0.05) was found for the litter inoculated with the higher level of microbial diversity. Higher respiration was also found for the younger litter compared to the older litter and both litter origins. This shows a reduction in microbial respiration with substrate age and inoculation diversity, suggesting that microbial diversity supports the decomposition of soil organic matter.

## 1. Introduction

In the global carbon cycle, the role of soil organic matter production and litter decomposition is very important [1,2,3,4,5]. Soil organic matter can enhance the structure of the soil and improve soil physical properties, and can be a nutrient source for biota living in the soil [6].

Litter decomposition is a key process in the carbon cycle in terrestrial ecosystems and can be performed by soil microorganisms, among other soil biota [7,8]. Plant litter represents a major source of organic matter entering soil [7,8]. Litter decomposition is a complex process. Litter has a very complex structure, both morphologically and chemically, and, as a consequence, a large group of diverse organisms typically cooperate in litter decomposition. There are substantial differences between individual plant species, which determine litter decomposition. Litter stoichiometry is a good predictor of litter decomposition; typically, litter with a low carbon-to-nitrogen (CN) ratio decomposes more easily than litter with a high CN ratio. Moreover, morphological structure and chemical composition of litter change with litter decomposition, with simple molecules that decompose first and structure elements of litter consisting of lignocelluloses and hemicelluloses, being the most persistent [7,8]. Consequently, microbial community composition also changes along this decomposition gradient, with each stage of decomposition being dominated by a specific microbial community adapted to specific conditions of a given decomposition stage [9,10,11]. These changes in microbial community composition and microbial diversity may change the rate of decomposition, even if the microbial biomass remains the same [11]. With modern techniques such as next-generation sequencing approaches, our knowledge about microbial diversity and community composition in key ecological processes has increased substantially [9,10,11,12,13,14]. Microbial diversity is very high and still largely unknown; thus, searching for simple descriptors of microbial community that affect key ecosystem processes is an important issue for contemporary research [11]. The presence of certain keystone microbial taxa essentially affects the functioning of microbial community [15]. Many studies have also shown that an increase in microbial diversity results in an increase in litter decomposition [16,17]. However, the relationship between microbial diversity and the rate of decomposition needs more research to be conducted [18,19,20].

Microbial diversity and the composition of the microbial community are also controlled by other factors, such as soil’s physical and chemical characteristics (soil pH and soil type), and environmental (humidity and temperature) and biological factors including litter quality (plant communities and other soil biota). In addition, changes in the environment caused by human activities such as the reclamation of sites also influence both microbial diversity and the microbial community [21,22,23]. The microbial community also changes during litter decomposition [24]. The microbial community is adapted to a certain stage of decomposition. However, there seems to be a high level of redundancy in the microbial community and the community can adapt to local conditions rather fast [25]. A high level of redundancy is also suggested by Angst et al. [26], who found a similar rate of decomposition in two systems with different microbial communities in parallel decomposition experiments.

Microbial diversity has often been reported to be positively correlated with microbial respiration, which is assumed to be a proxy for organic matter decomposition [27]. However, changes in microbial diversity vs. microbial biomass have seldom been addressed. Changes in microbial biomass might have a larger impact on microbial respiration than changes in microbial diversity.

In this study, we experimentally manipulated microbial diversity and community composition related to early and later stages of plant litter decomposition. We explored the question of whether microbial diversity per se, or rather adaptation to a particular stage of litter decomposition (a kind of home advantage), is a decisive factor driving microbial respiration, used here as a proxy for decomposition rate. We hypothesized that microbial diversity supports litter decomposition of soil organic matter, while adaptation of the microbial community to a particular stage of decomposition is less important. To see how these principles are general, we used litter of contrasting quality, including litter of an N-fixing tree (alder) with a low CN ratio, and litter of successional regrowth with a higher CN ratio.

## 2. Materials and Methods

### 2.1. Sampling, Preparation, and Experimental Setup

The experimental setup was similar to the inoculation experiment described in Frouz et al. [28], except that our tests were performed with a different microbial diversity and inoculum from leaf litter. The field part of the experiment took place in the extensively studied common garden experiment of the LTER site of the former Sokolov surface coal mine located in northwest Czech Republic (50.2417503 N, 12.7029267 E); its average annual precipitation is 650 mm and the mean annual temperature is 6.8 °C (Supplementary Figure S1). The area is covered by a mosaic of single-species forest patches, planted as a reclamation measure, and patches of unreclaimed sites where forest develops by its own natural processes in a way that individual tree patches are randomly spread over the heap. This gives a “common garden” experiment of a landscape dimension. In this common garden experiment, various tree species were planted at the same time, on the same substrate and experiencing the same climatic conditions. The spoil heaps were formed by Tertiary clays of the so-called cypris formation; its prevailing minerals were kaolinite, montmorillonite, and illite. When dumped on the heap, this Tertiary clay had an alkaline pH of 8, but at the time of this experiment, pH in both forest stands varied between 6 and 7.

More details about the sites, soil chemistry, and soil fauna communities can be found in Frouz et al. [28]. For our experiment, two forest stands were chosen: alder (*Alnus glutinosa*) plantations and succession sites overgrown by birch (*Betula pendula*), aspen (*Populus tremula*), and willow (*Salix caprea*). Both forest stands were about 40 years old at the time of this study. Alder, as a nitrogen fixer, provides litter with a low CN ratio (23), which decomposes fast, while leaf litter from succession sites had a higher CN ratio (31) and decomposes more slowly [28]. Freshly senescent leaf litter (hereafter called simply litter) from each of the forests was collected using litter traps. Three traps with an area of 50 × 50 cm were employed during the time of litter fall and collected biweekly. About 500 g of fresh litter was placed in litter bags with a mesh size excluding fauna (0.005 mm) and kept on the forest floor of the same forest stand where litter was collected. Litter bags were located on the interface between litter and mineral soil, and were in contact with the soil. Half of the bags were collected after three months and the other half after twelve months. After collection, the litter was freeze-dried. About 20 g was kept for future inoculation and the rest was sterilized with 27 kGy of γ-radiation.

**Figure 1:**
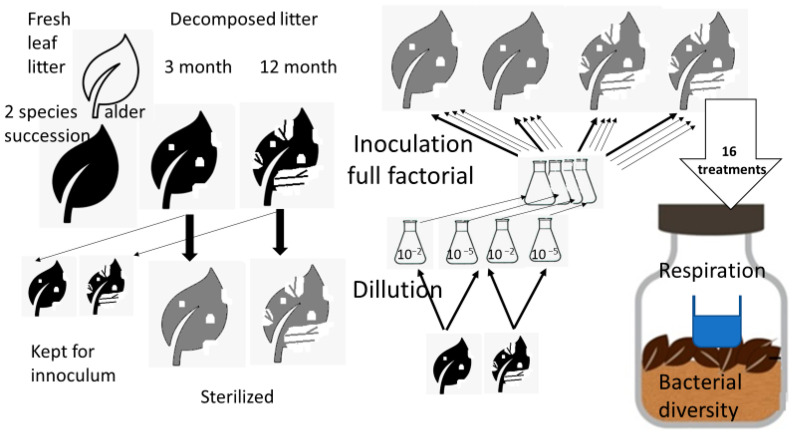
Scheme of the experimental design. The experiment was conducted with two types of litter; however, for simplicity, the workflow in the figure is depicted in detail only for one type of litter; for other types of litter it would be identical. Likewise, each combination of litter age and dilution was inoculated with sterilized leaf litter substrates of conspecific litter in both ages to obtain the full factorial combination for each litter type. This brings 2 sources of inoculate × 2 dilutions inoculated into 2 substrates of different ages, which means 8 treatments per litter type and 16 treatments in total. Each treatment was the subject of respiration measurement and bacterial diversity characterization.

Sterilized substrates were inoculated with a suspension of unsterilized leaf litter with dilutions of 10^−2^ and 10^−5^ to obtain different of microbial diversity levels, where a less diluted suspension supports higher diversity [29,30]. In this case, dilution rates were the proportion of leaf litter used to form the inoculum relative to the sterilized leaf litter (Figure 1). The stock solution of the inoculum was prepared by vortex shaking (2000 rpm) 2 g of non-sterilized leaf material in 10 mL of sterilized distilled water, followed by dilution as required with sterilized distilled water. The litter was inoculated with inoculum of the same litter species, but in both cases inoculum of both 3- and 12-month-old litter was added to 3- and 12-month-old litter in a full factorial manner. This procedure resulted in eight litter inoculum dilution combinations (treatments) coded as follows: 3-3-2, 3-3-5, 3-12-2, 3-12-5, 12-3-2, 12-3-5, 12-12-2, and 12-12-5, the first number indicating the age of the leaf litter substrate used (3—three-month old substrate, called young substrate or young litter hereafter; and 12—twelve-month old substrate, called old substrate or old litter hereafter). The second number indicates the litter from which the inoculation was drawn, and with which the sterile leaf litter was subsequently inoculated (3—three-month inoculum; 12—twelve-month inoculum). The third number indicates which dilution was used for each treatment (2—samples with a 10^−2^ dilution; 5—samples with a 10^−5^ dilution) (Figure 1). The experiment lasted for three months, after which the samples of all the treatments were analyzed.

### 2.2. Measurements

Respiration was measured weekly for three months. The amount of CO_2_ released was determined by means of titration [30]. The released carbon dioxide was initially absorbed in 0.5 M NaOH.

At the end of the experiments, material from individual treatments was pooled and used for characterization of the microbial community using phospholipid fatty acids (PLFAs) and next-generation sequencing. PLFAs were extracted from 1 g of lyophilized samples. The samples were extracted with a chloroform–methanol–phosphate buffer mixture (1:2:0.8). Lipids extracted from the sample were separated with solid-phase extraction cartridges (LiChrolut Si 60, Merck, Darmstadt, Germany). The samples were eluted from the cartridges in three fractions: the phospholipid fraction was eluted with 2 mL of methanol; the glycolipid fraction was eluted with 6 mL of acetone; and the neutral lipid fraction was eluted with 2 mL of chloroform [31]. Fractions were exposed to mild alkaline methanolysis at 37 °C for 15 min [32]. The free methyl esters of the PLFAs were analyzed [33] with gas chromatography and mass spectrometry (450-GC, 240-MS ion trap detector, Varian, Palo Alto, CA, USA). Fungal biomass was quantified based on the 18:2u6,9 content (PLFAF), and bacterial biomass was quantified as the sum of i14:0, i15:0, a15:0, 16:1u7t, 16:1u9, 16:1u7, 10Me-16:0, i17:0, a17:0, cy17:0, 17:0, 10Me-17:0, 10Me-18:0, and cy19:0 (PLFAB). The fatty acids found in both bacteria and fungi, 15:0, 16:0, and 18:1u7, were excluded from the analysis. The relative content of individual PLFA molecules was also calculated. The total content of all PLFA molecules (PLFAT) was used as a measure of total microbial biomass. The fungal/bacterial biomass (F/B) ratio was calculated as PLFAF/PLFAB. 

Bacterial community composition was assessed by 16S rDNA amplicon sequencing. Briefly, genomic DNA was extracted from samples as described by Sagová-Marečková et al. [34] and then amplified by PCR. For the bacterial community analysis, the V4 region of the bacterial 16S rRNA gene was amplified using the barcoded primers 515F and 806R as previously described [35]. PCR was performed in triplicate for each sample, and the resulting amplicons were purified and pooled, and libraries prepared using the TruSeq PCR Free Kit (Illumina, San Diego, CA, USA) were subjected to sequencing in house on the Illumina MiSeq platform (2 × 250 bp paired-end reads).

Sequence data were processed using SEED 2.0 [36] and USEARCH v 10.0.240 for Linux/Unix, following the suggested pipeline [37,38]. Briefly, all forward and reverse reads were merged, and the barcoded primers were removed from the reads. Operational taxonomical units (OTUs) were constructed by clustering sequences at a 97% similarity threshold using the UPARSE algorithm implemented in USEARCH10 with automatic chimera removal. For each OTU represented by the most abundant sequence, the closest hit at the genus level was identified using BLASTn database against the RDP database [39], and all nonbacterial sequences were removed. Before diversity estimation, numbers of reads per sample were randomly rarefied to 10,000 per sample.

### 2.3. Data Analysis

The obtained data were modified in Microsoft Office Excel and statistically evaluated in the Statistica program (TIBCO^®^, version 13.3; Software for Statistical Computation; USA). To assess respiration at different substrate and inoculation levels at their interactions for each litter category, and to classify the homogeneous groups and significant differences between treatments, we used three-way analysis of variance (ANOVA) followed by a Fischer LSD post hoc test. The interactions between and variability in treatments were visualized by means of principle component analysis (PCA, [40]). Nonlinear multidimensional scaling (NMDS) was used to illustrate the community variability in response environmental variables without direct testing of individual variables. Parameter stress indicating the discrepancy between the original distance matrix and distances is also displayed in the ordination diagram. NMDS was conducted with the Canoco 5 software [41].

## 3. Results

**Figure 2:**
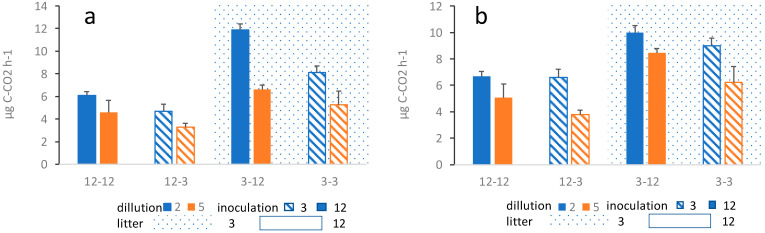
Average microbial respiration in (**a**) alder leaf litter treatments and (**b**) successional leaf litter treatments. The first number shows the age of the substrate (3-month or 12-month-old substrate), the second number shows one of the 3- or 12-month-old substrates from which the inoculum was drawn. Blue bars show the less diluted samples (10^−2^) and orange bars show the more diluted sample (10^−5^) while shagreen refer to inoculation source, hatched for three months old substrate solid for 12 months old substrate, in both dilutions, i.e., both colors. Corresponding three-way ANOVA results are summarized in Table 1.

The outcome of the overall average respiration during the course of the entire project is similar for both alder and successional litter. The respiration is significantly affected by age of previous litter decomposition, inoculation source, as well as dilution of the inoculum (Figure 2). In both cases, three-way ANOVA shows a significant effect of the source of the inoculum, where substrates inoculated with the inoculum originating from litter having been decomposing for 12 months show a significantly higher respiration than substrates inoculated with the inoculum originating from litter having been decomposing for 3 months (Table 1; Figure 2). Likewise, substrates inoculated with the less diluted inoculum show a significantly higher respiration (Table 1). Finally, respiration was significantly higher in three-month-old substrates compared to 12-month-old substrates, regardless of the inoculum (Table 1; Figure 2). The effect of these factors was similar in both alder and succession litter. However, in succession litter, there were some interactions between treatments (Table 1; Figure 2), which however do not affect the overall pattern of results. In alder litter, no interactions between treatments have been found. In all treatments, respiration gradually decreased during the three months of respiration measurements (Figure S1).

**Figure 3:**
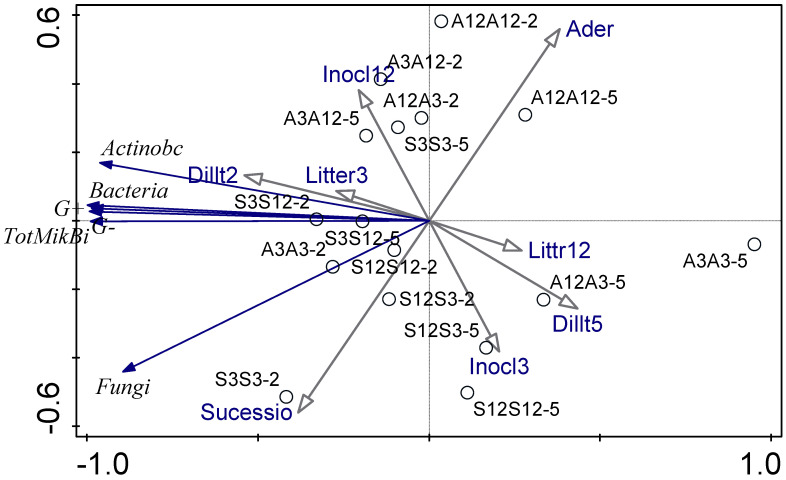
PCA analysis of microbial community studied using PLFA. The first letter shows the age of the substrate (old, 12-month substrate or young, 3-month substrate), the second letter shows the inoculation from one of young or old substrates. A is the alder litter treatment and S is the successional litter treatment. Microbial community parameters are depicted by blue arrows, environmental conditions are depicted by black arrows.

Figure 3 shows the relationship between the experimental parameters and how these interactions affect the microbial community studied by means of PLFA and decomposition rate using a PCA diagram. The first ordination axis explains 95.6% of the data variability and the second ordination axis explains 2.5% of the data variability. The first ordination axis corresponds with the gradient of total microbial biomass and biomass of most microbial groups. Microbial biomass is higher for treatments with less decomposed litter and for treatments inoculated by less diluted inoculum. The second ordination axis reflects differences between alder and successional litter. Successional litter seems to be associated with higher biomass of fungi. The second ordination axis is also affected by age of litter, which was the source of microbial inoculum. Alder treatments are less dependent on microbial communities and substrate or inoculation type and level (Figure 3). The fungal–bacterial ratio based on PLFA was low in all treatments. When we use RDA (ordination diagram, Supplementary Figure S2), substrate species, substrate age (decomposition time), inoculum decomposition age, and dilution as only explanatory variables, dilution was the only factor with a significant effect on microbial community composition evaluated using PLFA.

The highest operational taxonomic unit (OTU) richness was determined for the 12-12-2 treatment from successional covered litter and came close to the value in the 12-12-5 treatment from alder litter (Table 2). The lowest OTU richness was determined in the 3-3-5 sample, and a slightly higher value in the 12-3-5 sample, both from alder litter. The OTU values were always lower for the less diluted samples (10^−2^) and lower for alder compared to successional covered treatments (except for a few values; Table 2).

**Figure 4:**
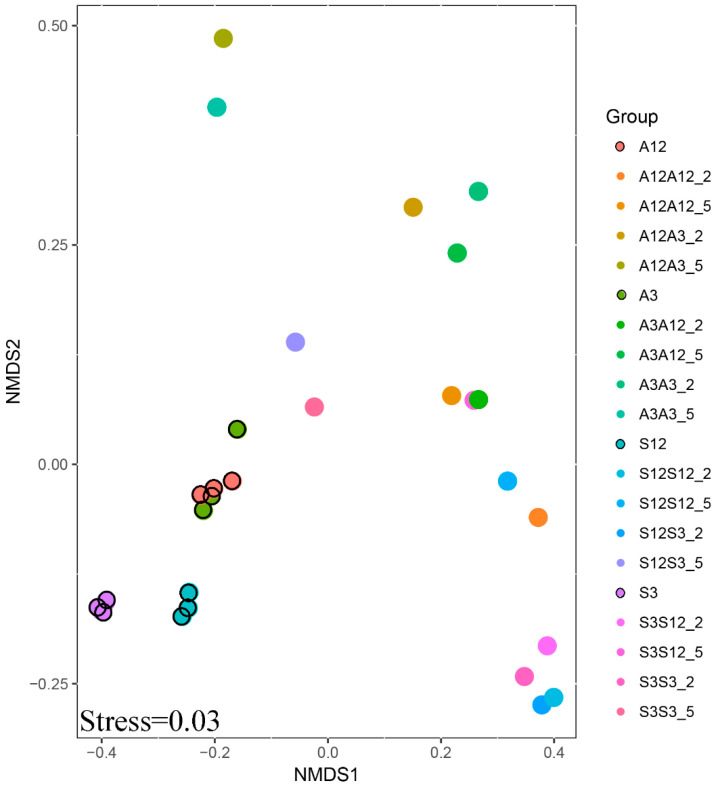
NMDS of the OTU composition of the microbial community. The first letter shows the age of the substrate (old, 12-month substrate or young, 3-month substrate); the second letter shows the inoculation from one of young or old substrates. A is the alder litter treatment and S is the successional litter treatment.

The interaction of experimental parameters was also confirmed by the results of NMDS analysis (Figure 4). More similarity is found in the samples from the successional treatments. A dense cluster of points can be observed for these treatments. One can also see that three separate samples taken from leaf litter used for inoculation were very similar to each other. Also, differences in bacterial community structure, between alder and succession litter, and between conspecific litter types in various stages of decomposition were smaller than differences between individual treatments caused by inoculation.

Proteobacteria and Actinobacteria are the most dominant bacterial phyla in all the samples. Proteobacteria dominate in original alder litter after 3 and 12 months of decomposition, as well as in all treatments inoculated in alder litter. Original successional litter was dominated by Actinobacteria. This applies to litter after 3 and 12 months of decomposition; however, all treatments inoculated on successional litter were dominated by Proteobacteria.

## 4. Discussion

In agreement with our hypothesis expecting that microbial diversity is a key driver of soil respiration and in agreement with some other authors [20,42,43], we found that microbial diversity significantly increases the decomposition rate. This was shown by applying two independent approaches related to the source and origin of the inoculum. Decomposition was significantly higher for the less diluted inoculum, which was more diverse, and also for the inoculum coming from litter having decomposed for 12 months, which was also more diverse. This effect was consistent for two types of leaf litter, which had very different quality, including litter from a nitrogen-fixing tree, which has a low CN ratio, and litter from successional stands with a higher CN ratio. This supports the idea that this observation should be broadly applicable to other types of broadleaf litter. On the contrary, we found no support for higher decomposition rates in the case when the inoculum originates from the matching decomposition stage. Thus, no support for home advantage was found. This suggests that the diversity of the inoculum rather than its composition affects the final decomposition rate. We suppose this is due to the fact that the microbial community is highly redundant and gets quickly adapted to local conditions [26]. A higher diversity increases the level of this redundancy, which may allow the microbial community to become quickly tailored to current environmental conditions. This is indirectly supported by the finding by Angst et al. [26], who found a similar rate of decomposition in two parallel decomposition treatments with different microbial communities. In addition to higher diversity in terms of OTU number, more diverse communities, namely those originating from less diluted inoculum, are also likely to contain more rare species, as rare species are more likely to be lost by dilution than common ones. This supports the idea that rare species may be essential for microbial community function.

We did not find any context dependency of diversity effect as observed by Vicena et al. [29]. This is likely due to the fact that the tendency in the previous study was caused by a shift between a fungal-dominated and a bacterial-dominated system, while in the latter study, all the communities seem to have been bacterial-dominated, based on PLFA results. Interestingly, the fact that diversity counts more than community composition is in contradiction to results obtained for non-microbial communities, such as in the case of the effect of soil fauna on decomposition rate, where community composition (namely, the presence of a major functional group) is typically more important that diversity per se [44]. This is likely due to the fact that redundancy in the microbial community is much higher than in the animal community, and also the rate at which the community can adapt to current conditions is faster, as generation time is much shorter in microbes than in soil fauna. This discrepancy between the effect of diversity per se vs. community composition between fauna and microbes clearly represents a promising question for future research.

In terms of inoculum dilution, there may be also an effect of microbial biomass, which is generally higher in less diluted inoculum, based on PLFA results; however, in both dilutions, as well as litter species, the effect is stronger for inoculum coming from more decomposed litter, which is more diverse, so we believe that diversity is more important in this case than biomass. In contrast to the observation of Frouz et al. [28], we do not see any deterministic effect of the substrate on the microbial community in sterilized and inoculated treatments; instead, the microbial community in these treatments is an interplay between the substrate and inoculum. This is likely due to the fact that the litter surface is spatially a much simpler matrix than soil, which makes extraction of the microbial community in inoculum, as well as the inoculation process, easier. Microbial communities in inoculated treatments are, however, much more diverse than microbial communities in original litter.

Besides contributing to our understanding of the relationship between microbial diversity and function, the observation that litter is colonized faster may also have practical consequences for the explanation of microbial activity during ecosystem restoration. In an earlier study conducted on a chronosequence of alder plantations and succession of all sites in the area of our experiment, it was found that decomposition rate increased with plot age [45]. In addition to other factors, this can be attributed to the fact that in early stages of soil development, litter falls on a soil surface formed by bare overburden with low microbial diversity [46]. Later, microbial diversity increased, in addition to a layer of partly decomposed litter and fauna feces produced from the litter accumulation in soil, which may also serve as an inoculum [46]. In other words, easier inoculation of fresh organic matter by a more diverse microbial community entering the soil may contribute to the observed increase in the decomposition rate during ecosystem development at post-mining sites, and probably also in other heavily disturbed areas.

Substrate quality affects the decomposition rate as well. In agreement with other authors [47,48,49], the composition rate decreases as the litter gets more decomposed. This is supported by two lines of evidence in our study. For one thing, litter that has been decomposing for three months in the field decomposes faster than litter that has been decomposing for 12 months, and at the same time, the rate of decomposition decreased with time in all treatments studied. This slowdown is likely to be caused by the fact that litter in the initial stages of decomposition contains a large amount of easily decomposable substances and, consequently, their decomposition by the microbial community is fast; but later, less decomposable substances prevail in litter and consequently decomposition slows down.

## 5. Conclusions

The present study demonstrates that the microbial diversity increases during litter decomposition, and that litter inoculation with a more diverse microbial community supports faster decomposition. On the other hand, inoculation with a microbial community coming from the same stage of litter decomposition as the inoculated litter does not speed up litter decomposition. In other words, microbial diversity is more important than the composition of the microbial community used for inoculation.

## Figures and Tables

**Table 1:** Output of three-way ANOVA testing of the effect of litter decomposition time (litter), source of inoculation (inoculation), which was litter at various decomposition times, and dilution of inoculum (dilution), *p* values for individual factors, and their interactions.

	Alder	Succession
1—litter	<0.001	<0.001
2—inoculation	0.047	<0.001
3—dilution	<0.001	<0.001
1 × 2	ns	0.042
1 × 3	ns	<0.001
2 × 3	ns	0.034

**Table 2:** Operational taxonomic unit (OTU) richness found in samples from alder or successional litter.

	3-3-2	3-3-5	3-12-2	3-12-5	12-12-2	12-12-5	12-3-2	12-3-5
OTU—alder litter	573	274	939	422	1067	1185	823	290
OTU—successional litter	998	572	1101	635	1197	714	1078	652

The first number shows the age of the substrate (3-month-old substrate or 12-month-old substrate), the second number shows one of the 3- or 12-month-old substrates from which the inoculum was drawn, the last number represent dilution 2 for 10^−2^ and 5 for 10^−5^.

## Data Availability

Data are available on request to corresponding author.

## Supplementary Materials

The following supporting information can be downloaded at: https://www.mdpi.com/article/10.3390/microorganisms13020351/s1, Figure S1: Picture of unreclaimed (a) and reclaimed (b) sites where litter was collected and where filed 15 litter bags were exposed, organization of laboratory incubation experiment (c), leftover of litter and 16 sand and laboratory litter bags used in laboratory respiration test; Figure S2: RDA ordination diagram using all environmental variables.
