# Supplementary material for: Microbial Diversity Drives Decomposition More than Advantage of Home Environment—Evidence from a Manipulation Experiment with Leaf Litter
Source: Microorganisms. 2025 Feb 6;13(2):351. doi: 10.3390/microorganisms13020351 (PMC11858187; doi:10.3390/microorganisms13020351)
Supplement: Supplementary file 1 [file microorganisms-13-00351-s001.zip › microorganisms-3403845-supplementary.pdf]

Aricle

# Microbial diversity drives decomposition more than advantage of home environment– evidence from a manipulation experiment with leaf litter

**Masoud M. Ardestani[1,2], Jaroslav Kukla[1], Tomáš Cajtham[1,3], Petr Baldrian[3], Jan Frouz[1,2,*]**

[1] Institute for Environmental Studies, Charles University, Benátská 2, 12801 Prague, Czech Republic
[2] Institute of Soil Biology and Biogeochemistry, Biology Centre of the Czech Academy of Sciences, Na Sádkách 7, 37005 České Budějovice, Czech Republic
[3] Institute of Microbiology of the Czech Academy of Sciences, Vídeňská 1083, 14200 Prague, Czech Republic
* Correspondence: author. Email: jan.frouz@natur.cuni.cz (Jan Frouz)

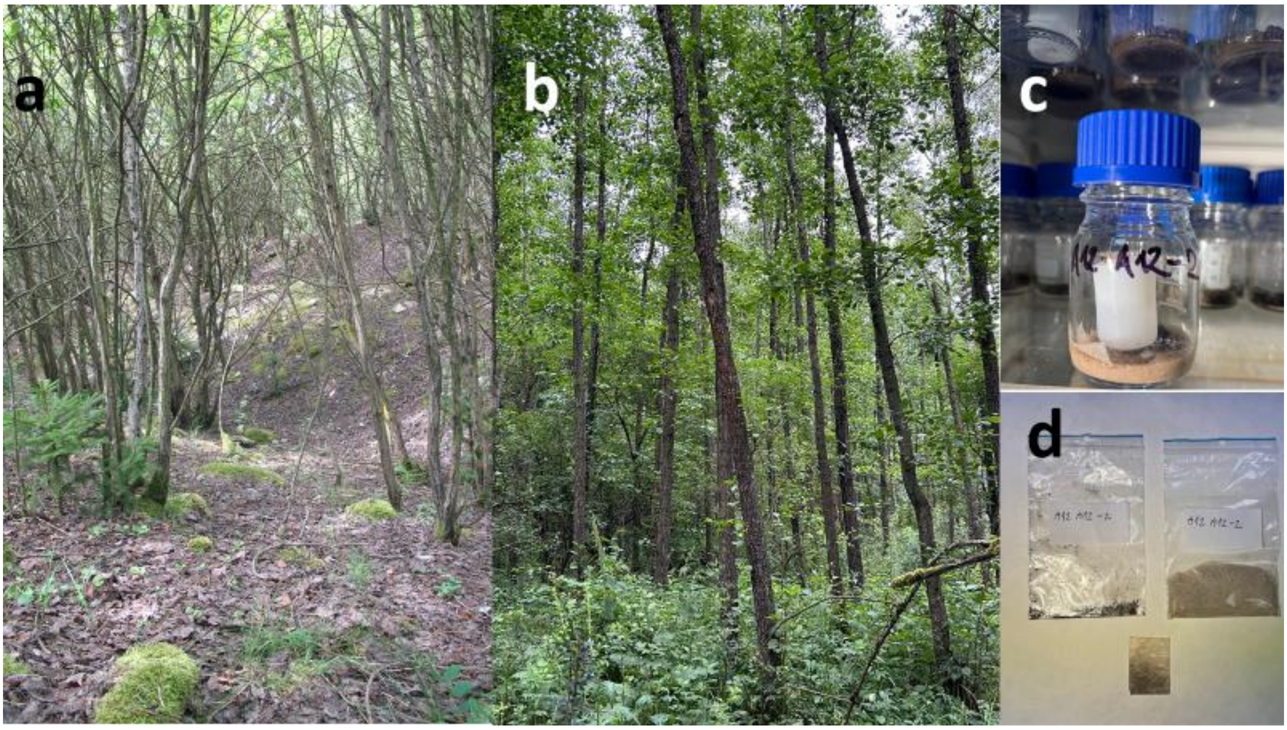


Figure S1 Picture of unreclaimed a) and reclaimed b) sites where litter was collected and where filed litter bags were exposed, organization of laboratory incubation experiment c) , leftover of litter and sand and laboratory litter bags used in laboratory respiration test

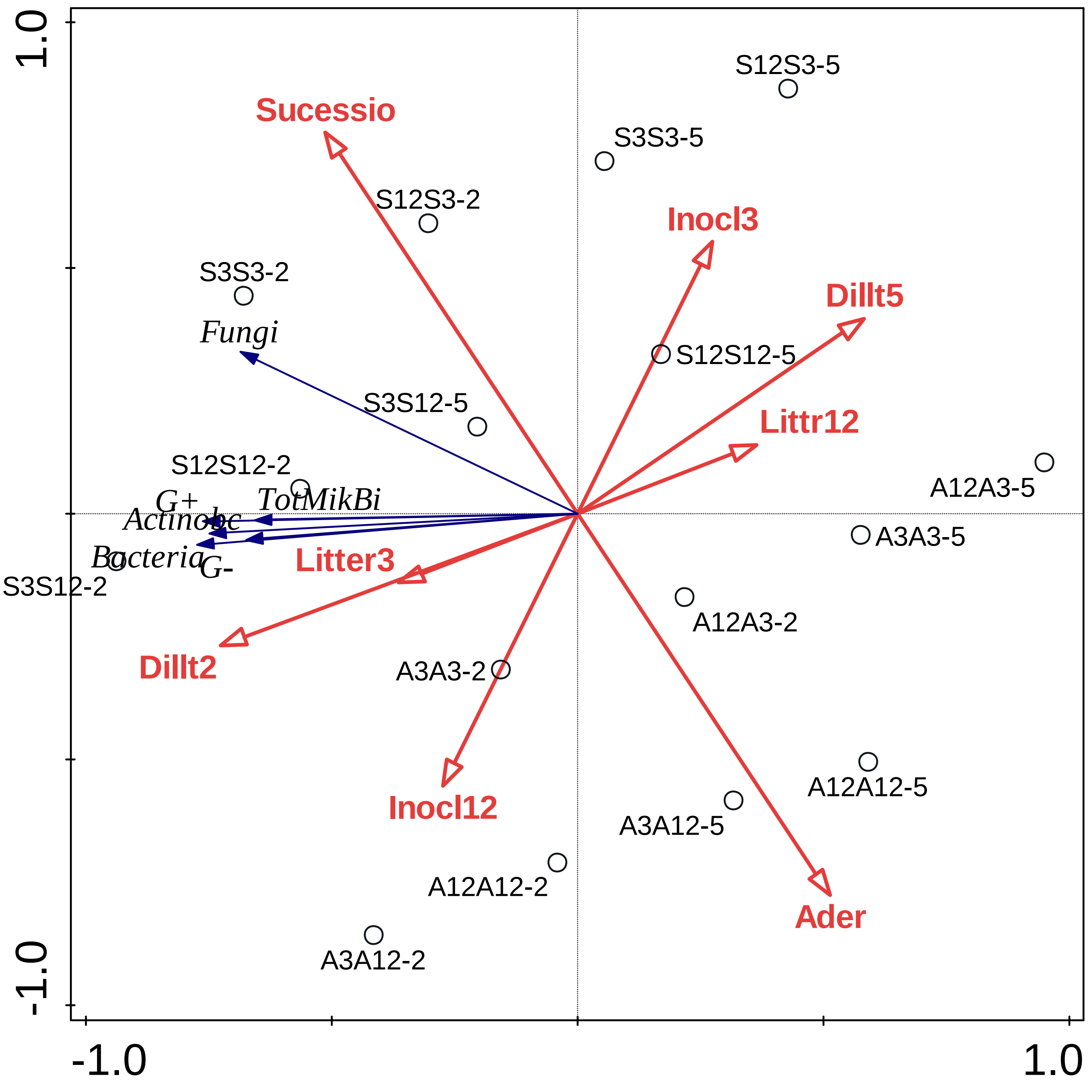


Figure S2 RDA ordination diagram using all environmental variables
